# Carbon nanotube-based multi electrode arrays for neuronal interfacing: progress and prospects

**DOI:** 10.3389/fncir.2012.00122

**Published:** 2013-01-09

**Authors:** Lilach Bareket-Keren, Yael Hanein

**Affiliations:** ^1^School of Electrical Engineering, Tel-Aviv UniversityTel-Aviv, Israel; ^2^Tel-Aviv University Center for Nanoscience and Nanotechnology, Tel-Aviv UniversityTel-Aviv, Israel

**Keywords:** carbon nanotubes, multi electrode array, neuronal recording, stimulation

## Abstract

Carbon nanotube (CNT) coatings have been demonstrated over the past several years as a promising material for neuronal interfacing applications. In particular, in the realm of neuronal implants, CNTs have major advantages owing to their unique mechanical and electrical properties. Here we review recent investigations utilizing CNTs in neuro-interfacing applications. Cell adhesion, neuronal engineering and multi electrode recordings with CNTs are described. We also highlight prospective advances in this field, in particular, progress toward flexible, bio-compatible CNT-based technology.

## Introduction

Extensive investigations over the past 50 years revealed the great potential of implanted electrodes for recording and stimulating neuronal signals. Such devices are currently being employed for the treatment of a wide range of conditions such as deafness, Parkinson's disease and chronic pain, to name just a few (Schwartz, 2004; Clark, 2006; Wichmann and DeLong, 2006; McCreery, 2008; Plow et al., 2012). Recent studies also suggested the use of neuro-stimulation in a growing number of additional disabling conditions, such as schizophrenia and Alzheimer's disease (George et al., 2007; Laxton et al., 2010). As high resolution, multi-site recording and stimulation devices are very attractive for neural recording and stimulation applications, the concept of multi electrode array (MEA) has gained increased attention in this field. A MEA device consists of an array of electrically conducting microelectrodes (typically 20–200 μm in diameter), connected to an external circuitry to allow recording or stimulation of neural electrical activity. Extensive effort has indeed demonstrated the potential of MEAs as an effective tool in various neurological applications. In particular, micro-fabrication technologies were employed to form finely shaped metallic [e.g., gold, platinum, and titanium nitride (TiN)] electrodes. The realization of such electrodes is readily achieved using a toolbox borrowed from the micro electro mechanical system (MEMS) field. This toolbox includes fabrication processes as well as materials with improved performances.

The scope of the current review is to explore, within the framework of micro fabricated neuro-electrodes, the employment of carbon nanotubes (CNTs) as a novel material with unique properties for neuro-applications. To this end, the CNT properties will be reviewed as well as their processing and fabrication into devices. The general field of micro fabricated neuro-electrodes will be introduced briefly and is beyond the scope of this review. We refer the reader for further reading on micro fabricated neuro-electrodes to HajjHassan et al. (2008), on the biocompatibility of CNTs to Warheit et al. (2004), Bottini et al. (2006), Carrero-Sanchez et al. (2006), and Firme and Bandaru (2010), and on the use of CNTs in biology to Bekyarova et al. (2005), Tarakanov et al. (2010), and Bottini et al. (2011).

We begin by reviewing the fundamental chemical, physical and electrical properties of CNTs (Thostenson et al., 2001; Harris, 2009; Lan et al., 2011). We then examine studies in which the neuron-CNT interface was explored. Next, the use of CNTs for neuronal patterning is discussed followed by a review of the electrical interfacing between CNTs and neurons and the study of CNT MEAs for neuronal applications. Finally, we discuss the progress toward flexible, bio-compatible CNT technology.

### Beyond conventional micro-fabrication

Despite a rapid recent development, contemporary MEAs for neuronal applications are still typified by relatively low signal to noise ratio (SNR), low spatial resolution (leading to poor site specificity) and limited biocompatibility. Clearly, further development is needed to make better electrodes suited for seamless integration between electronic devices and neuronal systems. The limited performances of these MEA systems stem primarily from the challenging interface between the biological systems and the artificial, electronic systems. The design of an interface between a living tissue and an electronic device must consider the dramatic structural and chemical differences between these two systems: Living tissues are soft, whereas electronic devices are usually rigid. Tissue conducts charges by ionic transport, whereas electronic devices conduct electrons and holes. Therefore, neural electrodes should accommodate differences in mechanical properties, bioactivity, and mechanisms of charge transport. Proper electrode-neuron interface is critical in ensuring both the viability of the cells and the effectiveness of the electrical interface.

A fundamental limiting feature of many contemporary MEAs is large electrode dimensions. Smaller electrodes would allow better spatial resolution and specific cell recording or stimulation. Also, reduction in electrode size (and therefore the dimensions of the entire device) is related to decreased tissue injury and immune response (Szarowski et al., 2003; Biran et al., 2005; Polikov et al., 2005; McConnell et al., 2009). While manufacturing small electrodes is technologically possible; the reduction in electrode size, needed for improving both stimulation and recording, is challenging. Small electrodes fail to provide sufficient charge injection owing to their high interface impedance. Low reversible charge storage capacity (CSC) means that the electrode cannot inject enough current to the tissue at small enough overpotential to avoid irreversible electrochemical reactions (i.e., electrolysis) and the ensuing damage to the electrode and the tissue (Cogan, 2008). Thus, to reduce electrode size without sacrificing the electrode ability to transfer charge, electrodes with high specific area are desired. High impedance also contributes to increased overall noise levels in recorded signals, thus reducing the recording sensitivity. An additional concern is the polarity of the electrode. For better biocompatibility, polar electrodes are desired (Merrill et al., 2005). These issues are further discussed later in the text.

Coupling neural cells intimately to the electrodes is also important otherwise the efficacy of both recording and stimulation are compromised. Recording is compromised by background noise of nearby neurons. Also, the conductance of the solution effects both recording and stimulation (Grattarola and Martinoia, 1993). The most common means to promote neural adhesion is through the use of cell adhesion proteins (Sorribas et al., 2001; Heller et al., 2005). Synthetic positively charged polymers, such as polylysine (Crompton et al., 2007) and poly(ethyleneimine) (PEI) (Ruardij et al., 2000) are commonly used to promote neural cell attachment (He and Bellamkonda, 2005; Khan and Newaz, 2010). The temperature sensitive Poly(N-isopropylacrylamide) (PNIPAm) was used to improve the binding between a retinal implant and the retina (Tunc et al., 2007). Conducting polymers (CPs), such as poly(ethylenedioxythiophene) (PEDOT), and polypyrrole (PPy) were used as neural growth substrate and electrode coating and are of particular interest due to their combined electronic and ionic conductivity (George et al., 2005; Abidian and Martin, 2008; Asplund et al., 2009; Abidian et al., 2010). The main disadvantage of CPs is their low stability under continued stimulation and exposure to ultra-violate (UV) radiation or heat. Applied voltage results with the insertion or removal of counter ions, so the CPs undergo swelling, shrinkage or breaking that gradually degrades their conductance (Yamato et al., 1995; Marciniak et al., 2004). Additionally, synthetic and CPs are often fabricated using complex or toxic polymerization schemes that are not well suited for cell interfacing applications. These residues are often not easily removed (Wan, 2008).

### Surface roughness and carbon nanotubes in neuronal interfacing

Recent studies have shown that surface topography is an important parameter affecting neuronal anchoring and branching (Seidlits et al., 2008; Hoffman-Kim et al., 2010; Roach et al., 2010). In fact, cells preferentially adhere to rough surfaces when exposed to the same chemistry (Fan et al., 2002). Therefore, new electrode materials were investigated to realize electrodes with improved electrical properties, affinity to neuronal cells and biocompatibility utilizing the electrode morphological properties rather than their chemical ones.

An ideal material to meet these requirements is CNTs. CNTs are well suited for neural electrical interfacing applications owing to their large surface area, superior electrical and mechanical properties, as well as their ability to support excellent neuronal cell adhesion (Malarkey and Parpura, 2007; Ben-Jacob and Hanein, 2008; Voge and Stegemann, 2011). Recent studies have indeed confirmed the great potential of CNT surfaces as a bio-compatible substrate on which neurons can readily adhere. This affinity was linked to surface properties including roughness, polarity, charge, and chemistry (Hu et al., 2004; Gabay et al., 2005a,b; Malarkey et al., 2009; Sorkin et al., 2009). CNT high surface area can lead to a significant increase in charge injection capacity and decreased interfacial impedance (Gabay et al., 2007; Keefer et al., 2008).

Investigations so far focused on several main themes: The effect of chemically modified CNTs on the viability of neuronal cells, process outgrowth and branching (Mattson et al., 2000; Hu et al., 2004; Matsumoto et al., 2007), electrical interfacing with neurons (Gheith et al., 2006; Wang et al., 2006; Gabay et al., 2007; Shein et al., 2009), and the development of neural implants (Webster et al., 2004; Nunes et al., 2012). CNTs are now widely investigated as an interfacing material for neuronal applications (Malarkey and Parpura, 2007; Ben-Jacob and Hanein, 2008; Pancrazio, 2008; Lee and Parpura, 2009; Voge and Stegemann, 2011). As highlighted above, both surface-chemistry and surface-topography are critically important parameters determining the formation of effective electrodes. Many schemes have been developed addressing these challenges using CNT coatings (pristine and chemically modified) offering exciting opportunities as will be further explored below.

## Carbon nanotubes

We begin our review with a brief overview of the physical properties of CNTs. CNTs are hollow cylinders formed in the shape of a rolled graphite sheet. Single walled CNTs (SWCNTs) are the simplest of these objects with a diameter ranging between 0.4 and 2.5 nm and lengths of up to a few millimeters. Multi walled carbon nanotubes (MWCNTs) are composed of a set of coaxially organized SWCNTs and are 2–100 nm in diameter while their length can vary from one to several hundred micrometers (Harris, 2009). The arrangement of the carbon atoms in the graphene sheet can be of different chirality: armchair, chiral, or zigzag. The chirality, as well as the tube diameter and the number of graphene walls, determine the CNT conductivity. Generally, SWCNTs can be metallic or semiconducting with MWCNTs featuring metallic behavior (Charlier et al., 2007). CNTs are also mechanically stable with very high tensile strengths and chemical inertness (Ciraci et al., 2004; Hayashi et al., 2007). CNTs are commonly synthesized from a catalyst by a variety of methods including: chemical vapor deposition (CVD), electric arc discharge and laser ablation (Thostenson et al., 2001; Seah et al., 2011). Their physical properties make CNTs a durable nanomaterial for biological applications, especially where a long lasting material is desired (e.g., scaffolds for support of cellular growth). Although the surface of CNTs is fundamentally inert, it can be readily functionalized with different polymers or bioactive molecules, such as peptides and proteins to improve their biocompatibility and bioactivity (Bekyarova et al., 2005; Yang et al., 2007; Lu et al., 2009; Bottini et al., 2011).

### Carbon nanotubes and neurons

The first investigations into the use of CNTs in neuro-interfacing applications focused on characterizing neuronal adhesion and proliferation on CNT coated surfaces. Mattson and co-workers were the first to discuss the use of CNTs as a substrate for neuronal growth (Mattson et al., 2000). The researchers grew embryonic rat hippocampal neurons on cover slips covered with PEI and MWCNTs. They found that pristine MWCNT substrates allowed neuronal attachment but did not support neurite branching as elaborate as that of cells cultured on PEI-coated coverslips. However, when MWCNTs were non-covalently functionalized (by physiosorption) with 4-hydroxynonenal (4-HNE), a molecule that promotes neurite outgrowth, large increases in the number of neurites per cell and in the overall neurite lengths were observed. This study demonstrated that MWCNTs can serve as a permissive substrate for neuronal cell adhesion and growth and that modifying MWCNTs with a biologically relevant molecule can be used to modulate neuronal growth and neurite outgrowth (Mattson et al., 2000).

The pioneering work of Mattson and co-workers was followed by a succession of studies aiming to further elucidate the observed effects. Hu et al. studied the effect of charge. Longer neurites and more elaborate branching were observed on positively charged CNT substrates (Hu et al., 2004). The charge of a MWCNT substrate was modified by functionalization with carboxyl groups, poly-m-aminobenzene sulfonic (PABS) acid or ethylenediamine (EN) to create negatively, zwitterionic or positively charged nanotubes, respectively. The number of neurites was counted depending on the nature of the nanotubes and their functionalization. Xie and co-workers determined that MWCNT mats functionalized with carboxyl groups are a permissive substrate for rat dorsal root ganglion (DRG) neurons growth, as confirmed by scanning electron microscopy (SEM) imaging. The researchers further suggested that the functional groups act as anchoring seeds enhancing neural cells and neurite adhesion (Xie et al., 2006).

**Figure 1:**
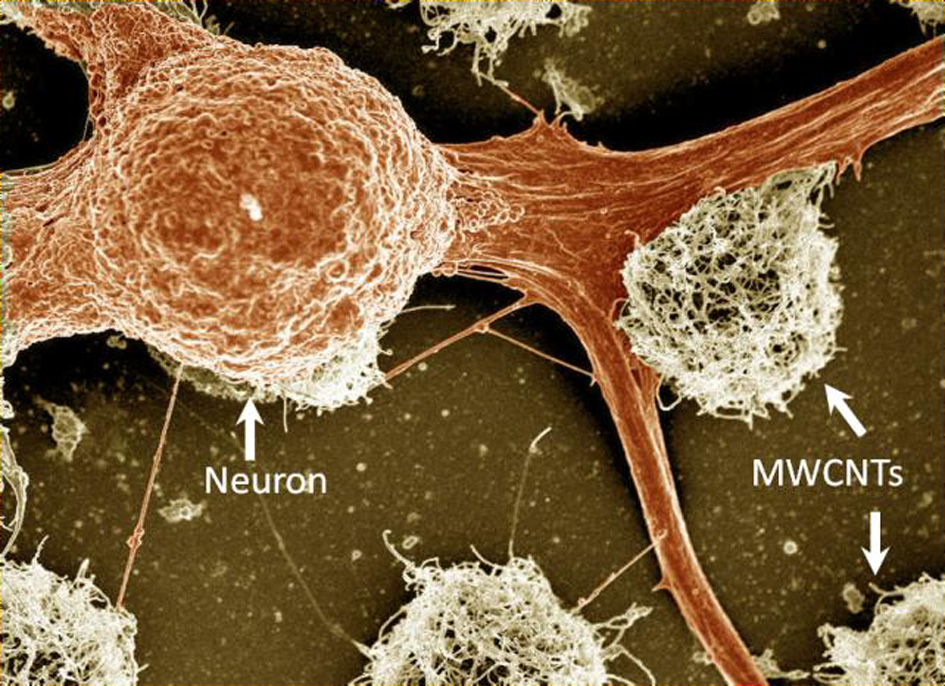
**A false-colored SEM image of fixed locust frontal ganglion neuronal cells cultured on carbon nanotube islands.** The carbon nanotube islands were grown using the chemical vapor deposition method directly on a quartz support. For further details see Sorkin et al. (2009). Width of field of view is 77 μm.

Covalent modifications of CNTs with neurotrophins, protein growth factors that promote the survival and differentiation of neurons, were studied by Matsumoto et al. (2007). MWCNTs were functionalized with nerve growth factor (NGF) and brain-derived neurotrophic factor (BDNF). Embryonic chick DRG neurite outgrowth on modified MWCNTs was similar to that seen with soluble NGF and BDNF in culturing media, indicating that the covalently attached factors were still bioactive. Pristine MWCNTs were also shown to support the growth of neurons (Gabay et al., 2005a,b; Galvan-Garcia et al., 2007). This effect is nicely illustrated in Figure 1 which shows the strong affinity between dissociated locust neurons and pristine CNT islands after several days of incubation. Galvan-Garcia and co-workers reported that MWCNTs in the form of sheets or yarns supported long-term growth of a variety of cell types ranging from skin fibroblasts and Schwann cells, to postnatal cortical and cerebellar neurons. When highly purified, these CNT sheets allowed neurons to extend processes in a similar number and length to those grown on planar polyornithine substrates (a permissive support). Thus, these results suggest that the interaction between neurons and CNTs may be affected by the purity of the CNTs, as well as by the three-dimensional organization of the CNT substrate.

Although initial investigations focused on MWCNTs, SWCNTs were also studied as neuronal substrates. Hu and co-workers synthesized a PEI functionalized SWCNT graft copolymer (SWCNT-PEI) (Hu et al., 2005). Covalent functionalization was used to turn SWCNTs to be soluble in aqueous media. Next, rat hippocampal neurons were cultured on coverslips coated with SWCNT-PEI and the results were compared with those of pristine MWCNT or PEI substrates. Fluorescent microscopy was used to examine neuronal viability, as indicated by their ability to accumulate the vital stain, calcein. It was found that SWCNT functionalization diluted the effect of the PEI's positive charge, resulting in neurite outgrowth and branching with intermediate extent to that of as-prepared CNT films or PEI alone. These results were consistent with the initial findings of Mattson and colleagues using fixed cells. Modified MWCNTs were found to be inferior to PEI as a culturing substrate (Hu et al., 2005). Gheith and co-workers demonstrated that freestanding SWCNT-polymer films prepared by the layer-by-layer (LBL) technique are compatible with neuronal cell culturing. The films were prepared by layering SWCNT with a negatively charged polyacrylic acid polymer. The SWCNTs were coated with amphiphilic poly (*N*-cetyl-4-vinylpyridinium bromide-*co*-*N*-ethyl-4-vinylpyridinium bromide-*co*-4-vinylpyridine). The presence of positively charged groups on the surface of this polymer promoted cell adhesion. Cell cultures of the neuronal model cell line NG108 effectively grew and proliferated on these substrates. Moreover, the number of neurites spun from individual cells exceeded those developed on traditional cell growth substrates (Gheith et al., 2005). However, not all CNT functionalization lead to the design of substrates that enhance neural cell growth. Liopo and co-workers showed that 4-tertbutylphenyl or 4-benzoic acid functionalized SWCNTs were less supportive of NG108 cell attachment and growth than pristine nanotubes (Liopo et al., 2006).

Carbon nanofibers (CNFs) are a form of carbon material closely related to MWCNTs and were also tested as a neuronal substrate. CNFs consist of multi-walled graphene structures stacked on top of each other like a stack of ice cream cones (Rodriguez, 1993). Nugen-Vu and co-workers directly grew forest-like vertically aligned CNFs (VACNFs) on a substrate with a lithographically patterned catalyst. After the CNF film was submerged in a liquid and dried, the CNFs irreversibly stuck together to form microbundles. A uniform freestanding film was achieved after coating the CNF with a thin layer of the CP PPy by electrochemical deposition. PC12 cell line grew as monolayers on the CNF films only after further coating with a collagen film. Otherwise cells appeared to float on top of the CNF surface (Nguyen-Vu et al., 2006). In a subsequent study, the neuronal marker NGF was introduced to the VACNF surface to promote the formation of well-differentiated cells with mature neurites. The freestanding VACNFs coated with PPy and NGF were found to bend toward the cell body and adhere to it. Therefore, it was suggested that the soft PPy coating contributes to better mechanical contact with cells due to a reduction in the local mechanical stress (Nguyen-Vu et al., 2007).

### CNT conductivity

Since CNTs may vary between being conducting and semi-conducting, their electrical properties were also studied. Malarkey and co-workers varied the conductivity of SWCNT-polyethylene glycol (PEG) graft copolymer coatings by changing the film thickness, while maintaining a constant surface roughness (Malarkey et al., 2009). Rat hippocampal neurons were then seeded. It was shown that thinner, less conducting SWCNT films, resulted in longer neurite processes, while thicker, more conductive films, produced larger cell bodies. Smooth, positively charged PEI substrates resulted in a larger number of growth cones per cell body. This study demonstrated that differences in conductance, roughness, and surface charge can modulate neuronal cell growth and morphology.

### Carbon nanotube surface roughness

Overall, the origin of the neuron-CNT interaction appears to be strongly affected by surface roughness. It was suggested that the roughness of CNTs contributes to anchoring neural cells (Zhang et al., 2005; Xie et al., 2006; Sorkin et al., 2009). Zhang et al. (2005) fabricated patterned vertical MWCNT surfaces. CNTs were then functionalized with poly-L-lysine (PLL). Cell cultures of the neuronal cell line H19-7 preferentially adhered to the MWCNT patterns. Neuronal growth cones were found to make contact with the nanotube surface, and these strong interactions allowed the neurons to spread along patterns and form interactions with one another. It was established that guided neurite growth was formed preferably on long vertical MWCNTs compared to short ones. This behavior was attributed by the authors to a possible increased adsorption of the PLL molecules onto the long nanotubes. Additional mechanism may be that long nanotubes are flexible and undergo deformation to accommodate the proliferating neurites.

**Figure 2:**
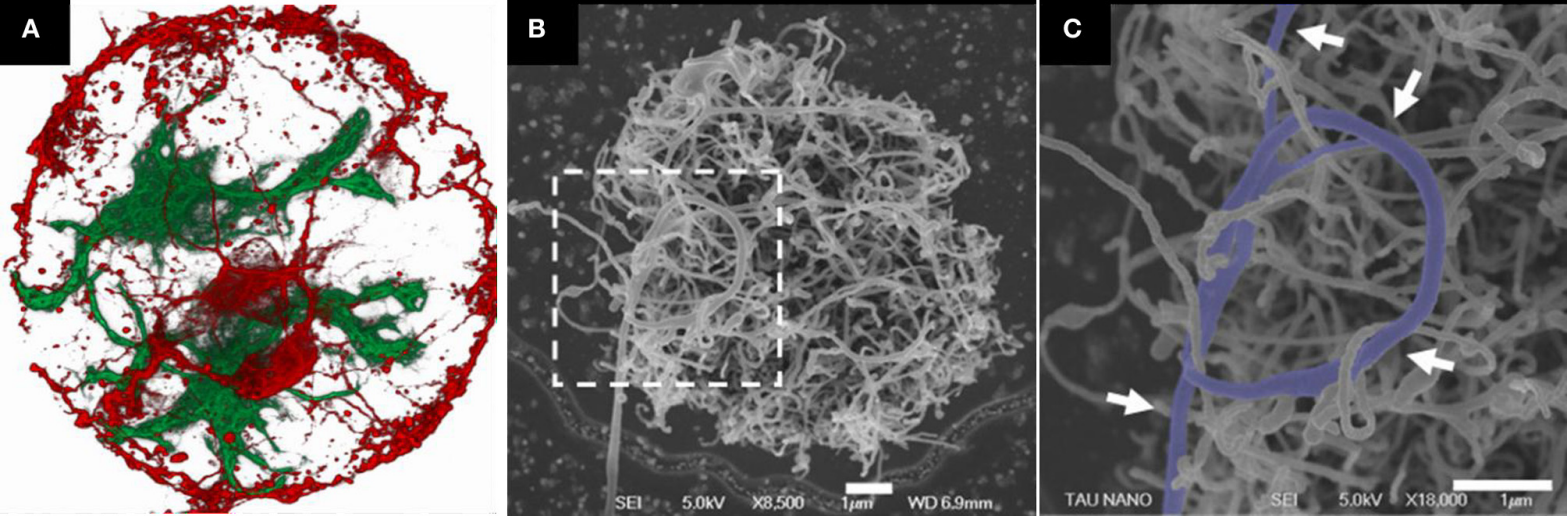
**Rat neuronal cultures on CNT islands. (A)** Fluorescent confocal image of fixed neurons (red) and glia cells (green) cultured on a carbon nanotube island. Disk diameter is 20 μm. **(B,C)** HRSEM images of a neuronal process forming a loop around several CNTs (designated by arrows). The image in **(C)** corresponds to the area marked by the dashed box in **(B)**. Clearly identifiable process segments were manually highlighted. Processes appear to bind to the carbon nanotube surface in a manner akin to that of tendrils. Adopted from Sorkin et al. (2009).

Sorkin and co-workers characterized the arrangement of neurons and glia cells on CNT surfaces (see Figure 2). Three-dimensional, small, isolated and pristine CNT islands were fabricated and plated with cells. Two biological model systems were used: cortical neurons from rats, and ganglion cells from locusts. Neurons were found bound and preferentially anchored to the rough surfaces. For both model systems, the morphology of neuronal processes on the small, isolated islands of high density CNTs was found to be conspicuously curled and entangled. In this study, it was demonstrated that the roughness of the surface must match the diameter of the neuronal processes in order to allow them to bind. It was suggested that entanglement, a mechanical effect, may constitute an additional mechanism by which neurons anchor themselves to rough surfaces (Sorkin et al., 2009).

Table 1 summarizes the main results described above, emphasizing how different CNTs and CNT modifications affect neuronal adhesion. Overall the general picture that emerges from these investigations is that MWCNTs, SWCNTs, and CNFs are permissive substrate for neuronal growth and proliferation. Neuronal interaction with CNTs is affected by CNT surface chemical modification, conductivity, charge, and roughness. Positive charge had a positive effect on neurite branching and length. Altering conductivity resulted with morphological changes in neurite length and cell body size. Surface roughness contributed to anchoring neurons to the surface. Chemical modifications of the CNT surface with 4-HNE, and PEI had a positive effect on neurite branching and growth, whereas modification with 4-tertbutylphenyl and 4-benzoic acid modified substrate diminished neuronal cell growth.

**Table 1:** **Neuronal adhesion on CNT coated surfaces**.

	**References**	**CNT surface**	**CNT modification**	***In vitro* bio-testing scheme**	**Results**
**MWCNTs**
	Mattson et al., 2000	MWCNTs coated cover slip	Pristine	Embryonic rat hippocampal cultures	Neuronal growth
			4-HNE		Increased neurites branching and length on modified CNTs
	Hu et al., 2004	MWCNTs coated cover slip	COO	Rat hippocampal cultures	Increased neurites branching and length on positively charged CNTs
			PABS		
			EN		
	Matsumoto et al., 2007	MWCNTs soluble in growth medium	NGF or BDNF	Embryonic chick DRG neurons	CNT attached factors and soluble factors had similar effect on neurite outgrowth
	Gabay et al., 2005a,b	MWCNT islands directly grown on catalyst patterned substrate	Pristine	Rat cortical cultures	Neuronal aggregation on CNT islands and formation of a neurite interconnected network
	Galvan-Garcia et al., 2007	Directionality oriented MWCNT sheets and yarns	Pristine	Rat shwan cells Mice primary cortical and cerebral neurons Mice DRG neurons	Multiple cell types permissiveness Neuronal interaction with CNTs may be affected by CNT purity and 3D structure
	Zhang et al., 2005	Vertical MWCNT arrays directly grown on catalyst patterned substrate	PLL	H19-7 cell line	Preferred guided neurite growth on longer flexible MWCNTs
	Xie et al., 2006	MWCNT mats	COOH	Rat DRG neurons	Longer neurites on modified CNTs Neurites intertwined with CNTs
	Sorkin et al., 2009	MWCNT islands directly grown on catalyst patterned substrate	Pristine	Rat cortical cultures Locust ganglion cells	Neurite morphology on high density CNT islands was curled and entangled
**SWCNTs**
	Hu et al., 2005	SWCNT coated cover slip	Pristine	Rat hippocampal cultures	Increased neurite outgrowth and branching on modified CNTs
			PEI		
	Gheith et al., 2005	SWCNT-PAA coated cover slip (by LBL)	Amphiphilic polymer	NG108 cell line	Neuronal growth
	Liopo et al., 2006	SWCNT coated PET film	4-tertbutylphenyl	NG108 cell line Rat primary peripheral neurons	Decreased neurites branching and length on modified CNTs
			4-benzoic acid		
	Malarkey et al., 2009	SWCNT coated cover slips	PEG	Rat hippocampal cultures	Longer neuritis on less conducting films Larger cell bodies on more conductive films
**CNFs**
	Nguyen-Vu et al., 2006	VACNFs directly grown on an catalyst patterned substrate	PPy	PC12 cell line	Soft PPy contributes to better mechanical coupling between cells and CNTs
	Nguyen-Vu et al., 2007		PPy and NGF		

### Carbon nanotubes for neuronal patterning

Patterned CNT films, such as those discussed above, provide a unique scheme for creating and studying engineered neuronal networks. Studies using patterned CNTs can provide insight into the collective activity of neural networks. CNT patterns also offer a route for developing three-dimensional scaffolds as a step toward designing circuits for bio-computational purposes and neuro-prosthetics applications. This approach can also be used to build advanced neuro-chips for bio-sensing applications (e.g., drug and toxin detection) where the structure and stability of the networks are important.

Zhang and co-workers cultured neurons on micron-scale patterns with different geometries. These patterns were designed to support an investigation into mechanisms underlying neuronal extension, guidance, and interaction. Straight lines, squares and circular features were used, as well as different lengths of the nanotubes. It was found that neurons preferentially adhered to MWCNT patterns. Growth cones were attached to the nanotube surface, allowing the neurons to spread along patterns and interact with one another (Zhang et al., 2005).

**Figure 3:**
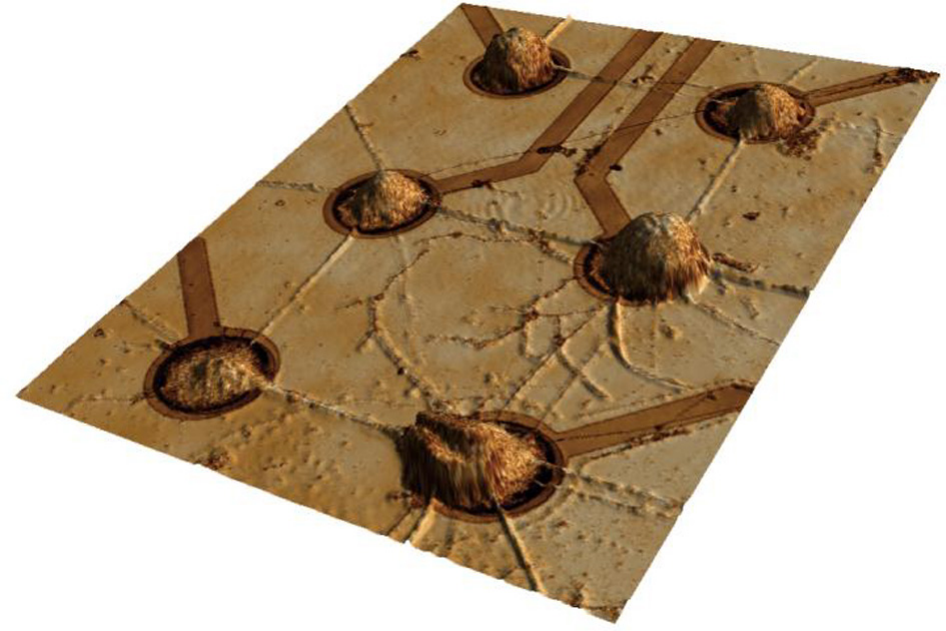
**A neuro-glia cortical culture from embryonic rats grown on a carbon nanotube micro electrode array.** Clusters of cells self-organized during culture development to position themselves on the electrodes. The distance between electrodes is 200 μm. Image acquired using a 3D confocal microscope (Shein et al., 2009).

CNT islands were also used extensively by us to engineer neuronal networks into a system with well-defined geometry (see Figure 3), so the interplay between geometry and neuronal activity can be systematically investigated (Gabay et al., 2005a,b; Sorkin et al., 2006, 2009; Greenbaum et al., 2009; Shein et al., 2009) (see Figure 2 for a typical example). In one of the first publications to use MWCNTs for neuronal interfacing applications, Gabay and co-workers imprinted a pattern of iron nanoparticle catalyst on quartz substrates using a poly (dimethylsiloxane) (PDMS) stamp and then grew CNTs from the iron catalyst islands. Rat cortical neurons and glial cells accumulated preferentially on the MWCNT islands and formed interconnected networks, bridging across the non-permissive quartz to form connections between adjacent islands. Using the patch clamp technique, cultured neurons were found to be electro-physiologically active with normal resting membrane potentials, demonstrating that the MWCNT did not alter the neuronal integrity (Gabay et al., 2005a,b).

In a successive work, Sorkin and co-workers examined the dynamics of neuronal network organization by placing rat cortical and hippocampal neurons on patterned MWCNT or poly-D-lysine patterned substrates. Cell clusters were found to spontaneously anchor to patterned islands with neurites, connecting nearby islands through a single non-adherent straight bundle composed of axons and dendrites. Square, triangular and circular structures of connectivity were successfully realized. Monitoring the dynamics of the networks in real time revealed that the self-assembly process is mainly driven by the ability of the cells to move while continuously stretching neurite bundles in between. The patterned networks were stable for as long as 11 weeks (Sorkin et al., 2006). In a subsequent study, Sorkin and co-workers cultured rat cortical neurons, as well as locust frontal ganglion neurons on micro-patterned MWCNT islands. Neuronal processes tended to wrap and entangle with the rough MWCNT islands. It appears that the similar dimensions of the CNTs (within the island) and the neurites supports an anchoring mechanism allowing neurons to attach (Sorkin et al., 2009). Greenbaum and co-workers demonstrated the use of specially designed CNT substrates to form small networks of locust frontal ganglion neurons. It was suggested that mechanical tension is created along the cell's processes and pulls the cell's soma; neuronal activity was recorded from single cells (Greenbaum et al., 2009). These effects were further explored (Anava et al., 2009; Hanein et al., 2011) to show that indeed mechanical effects are ubiquitous in these developing networks.

## Carbon nanotubes for electrical neuronal interfacing

As discussed in the “Introduction” section, contemporary electrodes used for neuro-prosthetic applications have relatively high impedance and poor CSC. In order to better appreciate these challenges and to evaluate CNTs potential in neuronal electrode applications, we begin with a brief overview of the electrical processes taking place at the neuron-electrode interface.

### Extracellular recording and stimulation of neuronal activity

Signal transmission in neuronal systems is the result of ionic currents passing through specific ion channels across the cell membrane. Extracellular recording methods monitor the electrical field associated with this dynamic. The time course of the extracellular action potential is typically ~1 ms and the amplitude is in the range of a few tens to a few hundreds of microvolts (Cogan, 2008; Buzsaki et al., 2012). This amplitude is significantly smaller than the corresponding intracellular spike, which is in the tens of millivolt range. Additionally, extra cellular signals diminish rapidly as a function of distance from the cell. A reverse process takes place during stimulation; charges are delivered from the electrode and induce a buildup of membrane potential. Under a strong enough field, voltage sensitive ions in the cell membrane trigger the generation of an action potential (Roth, 1994; Tehovnik, 1996; Basser and Roth, 2000).

Stimulating neurons and recording extracellular signals can be achieved using a conducting electrode placed close to the cell or its processes. The electrode electrochemical properties are fundamental to its performances as a stimulating or recording electrode. Clearly, an effective interface is a prerequisite for both stimulation and recording. While neuronal stimulation and recording are related in nature, these two applications have somewhat different requirements. Foremost, the amount of charge required for stimulation is orders of magnitude higher than what is recorded. Recording may often be impossible with electrodes which are well suited for stimulation. In neuronal recording, the typically small signals make noise considerations very important (Musial et al., 2002). For safe stimulation purpose, however, delivering the appropriate charge to the tissue without causing electrode or tissue damage is the main consideration (McCreery et al., 1988, 1990; Cogan, 2008).

**Figure 4:**
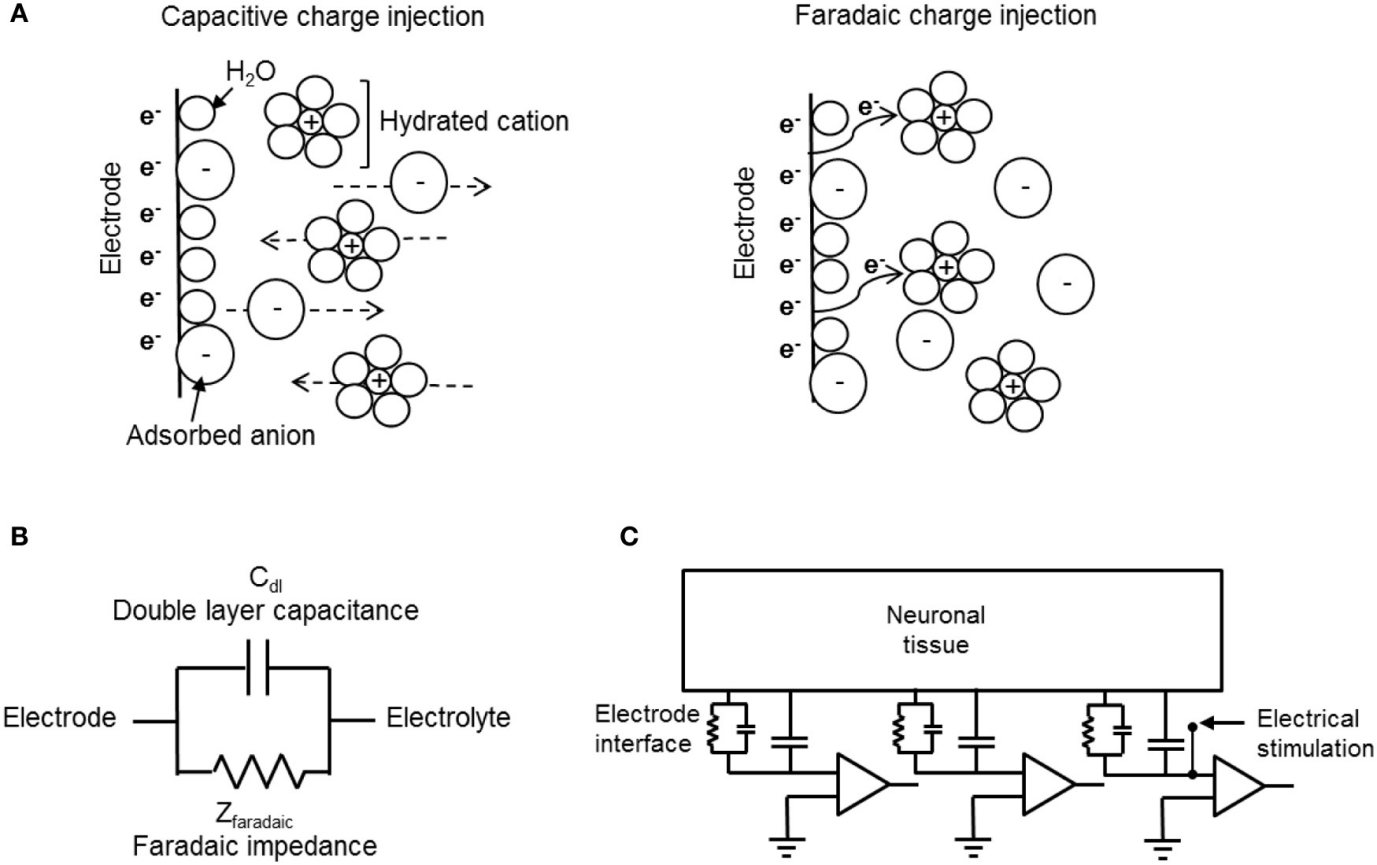
**Electrode-electrolyte interface and charge injection. (A)** Schematic representation of capacitive (left) and Faradaic (right) charge injection mechanisms. While capacitive charge injection includes redistribution of charge in the electrode-electrolyte interface, Faradaic process includes transfer of electrons. **(B)** An electrical circuit model for mechanisms of charge transfer at the electrode-electrolyte interface. **(C)** A circuit model for extracellular recording and stimulation from neuronal tissue using a MEA linked to external amplifiers. The model demonstrates the electrochemical interface resistance and capacitance of the CNT electrode and the solution derived shunt capacitance as well as the point of stimulation.

The electrode material and the reactions at the electrode-tissue interface (the reactions mediating the transition from electron flow in the electrode to ion flow in the tissue) are the main parameters determining the safe range for stimulation. The reactions taking place during charge injection can be capacitive or Faradaic (Figure 4A). Capacitive reactions involve displacement current and are associated with the charging and discharging of the electrode-electrolyte double layer due to redistribution of charged species in the electrolyte. Faradaic reactions, on the other hand, involve the transfer of electrons across the electrode-electrolyte interface and require that some species, on the surface of the electrode or in solution, are oxidized or reduced. These reactions can lead to irreversible processes that cause electrode or tissue damage. Therefore, while maximizing the current injected through an electrode is important, it has to be achieved ideally by using non-Faradaic electrodes. Capacitive charge delivery is therefore a critical consideration in the design of electrodes both for recording and stimulation.

The capacitive and Faradic reactions at the electrode-electrolyte are modeled by a simple electrical circuit consisting of two elements, a capacitor and a resistive element in parallel. Figures 4B,C illustrate circuit models of electrode-electrolyte interface and extracellular recording and stimulation of neuronal tissue, respectively. The capacitive mechanism, which represents the ability of the electrode to cause charge flow in the electrolyte without electron transfer, is modeled as a simple electrical capacitor called the double layer capacitor (Bard and Falkner, 2000; Merrill et al., 2005). Faradaic processes are modeled as a Faradaic impedance (Bard and Falkner, 2000; Merrill et al., 2005). There are two limiting cases derived from this model: The ideally polarizable electrode, and the ideally non-polarizable electrode (Bard and Falkner, 2000; Merrill et al., 2005). The ideally non-polarizable electrode has a zero Faradaic resistance, therefore current flows readily in Faradaic reactions and there is no change in voltage across the interface upon the passage of current. Thus, the electrode potential remains near equilibrium, even upon current flow. The ideally polarizable electrode has infinite Faradaic impedance element and is modeled by a pure capacitor. In an ideally polarizable electrode, all the current is transferred through capacitive action, thus the electrode potential is easily perturbed away from the equilibrium potential. Real electrode interfaces are modeled by the double layer capacitor in parallel with finite Faradaic impedance, together in series with the solution resistance. A highly polarizable electrode is one that can accommodate a large amount of injected charge on the double layer prior to initiating Faradaic reactions. Thus, for improved biocompatibility, highly polarizable electrodes are desired. An additional important parameter used is the description of neuronal stimulation electrodes is the reversible CSC, also known as the reversible charge injection limit (Robblee and Rose, 1990; Merrill et al., 2005). The CSC of an electrode is the total amount of charge that may be stored reversibly, including storage in the double layer capacitance, pseudocapacitance, or any reversible Faradaic reaction. The material used for the electrode, the size and shape of the electrode, the electrolyte composition, and parameters of the electrical stimulation waveform, all influence the CSC. We refer the reader for a detailed description of the electrochemical electrode-electrolyte interface of recording and stimulation neuronal electrodes (Bard and Falkner, 2000; Merrill et al., 2005; Cogan, 2008).

Overall, increased capacitance results in decreased impedance, and reduction in noise levels, as well as allowing wider voltage windows for safe electrical stimulation. Contemporary Faradaic electrode materials include mainly noble metals such as gold, platinum, titanium, and iridium, as well as alloys of these metals, iridium oxide, stainless steel, and highly doped semiconductors such as silicon. Capacitive electrode materials include TiN, tantalum-tantalum oxide, and the more recently investigated CNTs. The capacitive nature of CNT electrodes is therefore yet another major advantage.

### Carbon nanotubes for recording and stimulation of neuronal activity

As we discussed above, CNTs have several fundamental properties which make them ideally suited for neuronal interfacing. They support neuronal proliferation, they are conducting and they form extremely high specific area, capacitive electro-chemical electrodes. Accordingly, many recent studies have employed CNTs as a coating material for neuro-electrodes.

Direct stimulation of isolated neurons in culture using SWCNT coated substrate was demonstrated recently by several groups (Gheith et al., 2006; Liopo et al., 2006; Mazzatenta et al., 2007). Gheith and co-workers incorporated positively charged SWCNTs and poly acrylic acid into LBL multilayers with sufficiently high electrical conductivity to electrically stimulate a model neuronal cells line (NG108). The use of the SWCNT LBL films as culturing substrates did not perturb the key electrophysiological features of the NG108 cells, which confirms previous observations (Gheith et al., 2006). The electrical coupling of NG108 cells, as well as rat primary peripheral neurons to unmodified, as well as 4-tertbutylphenyl or 4-benzoic acid modified SWCNTs deposited onto polyethylene terephthalate (PET) films, were assessed by Liopo et al. (2006). Neurons showed voltage activated currents when electrically stimulated through the conducting SWCNT film. The same issue was subsequently addressed by Mazzatenta and co-workers who used electrophysiological measurements and computational modeling in order to understand the nature of the electrical coupling between neurons and pure SWCNTs (Mazzatenta et al., 2007). The authors cultured rat hippocampal neuronal on glass cover slips coated with pristine SWCNT films. SEM revealed contacts between neuronal membranes and SWCNTs. Electrical recordings using a patch clamp indicated that neurons grown on SWCNT substrates displayed spontaneous electrical activity. Stimulation of cultured neurons was achieved by applying current through the nanotube substrate. Finally, a mathematical model describing the electrical coupling between the SWCNT and the neurons was suggested (Mazzatenta et al., 2007).

Some studies suggested that CNTs boost neuronal electrical activity (Lovat et al., 2005; Cellot et al., 2009). Lovat and co-workers functionalized CNTs with pyrrolidine groups. This functionalization removed impurities and improved the CNT solubility in organic solvents. Glass cover slips were then coated with a drop of the solution. Evaporation of the solvent and heat treatment resulted with defunctionalization, leaving purified MWCNTs on the glass. Neurons grown on MWCNT films showed a six-fold increase in the frequency of the spontaneous postsynaptic currents and spontaneous action potential generation when compared to those grown on untreated glass. The authors proposed that the high conductivity of the CNT substrate might have affected the voltage-dependent membrane processes resulting in the increased activity (Lovat et al., 2005). Cellot and co-workers have suggested that CNTs improve electrical communication between neurons through the formation of tight contacts with the cell membranes. They used thin CNT films formed by solution deposition on glass followed by thermal treatment. Rat hippocampal neurons were seeded onto the films and showed an increase in synaptic firing (Cellot et al., 2009), enhanced formation of synapses as well as changes in synaptic dynamics (Cellot et al., 2011).

Composite CNT coatings enhance recording and stimulation of neurons *in vitro* and *in vivo* by decreasing the impedance and increasing charge transfer. Keefer and co-workers successfully coated electrodes with MWCNTs using different deposition schemes (Keefer et al., 2008). Commercial tungsten and stainless steel sharpened wire electrodes were coated with CNTs, using covalent attachment of the CNT coating, electrodeposition of CNT-gold coating or electrodeposition of CNT combined with CP (PPy). The different CNT coatings resulted with lower impedance and higher charge transfer capacity compared with bare metal electrodes. *In vivo* recording quality of CNT-coated sharp electrodes was tested in the motor cortex of anesthetized rats and in the visual cortex of monkeys. Compared with bare metal electrodes, CNT coated electrodes had reduced noise and improved detection of spontaneous activity (Keefer et al., 2008). Baranauskas and co-workers tested PPy-CNT coated platinum/tungsten microelectrodes. PPy-CNT coating significantly reduced the microelectrode impedance and induced a significant improvement of the SNR, up to four-fold on average. *In vivo* signals were recorded from rat cortex (Baranauskas et al., 2011). Other CPs-CNT composite coatings including PPy-CNT (Lu et al., 2010; Chen et al., 2011a) and PEDOT-CNT (Luo et al., 2011) were tested. These coatings similarly resulted with enhanced electrochemical properties and were found bio-compatible. The devices were not used in recording or stimulation. The PPy-CNT coatings highly improve the electrochemical performance of the test electrodes and further investigation into the durability of these coatings under long-term stimulation and recording use would be important to reveal their full potential.

Collectively, the studies reviewed above show that CNTs may provide a superior mean for electrical coupling between devices and neuron. We shall now discuss the use of CNTs electrodes for both electrical recordings and stimulation of neurons in the form of MEAs.

### Carbon nanotube MEA for neuronal recording and stimulation

A major development in the use of CNT in neuro-applications is the design and fabrication of CNT MEAs (Gabay et al., 2007). Such MEAs were made by synthesizing islands of high density CNTs. Both MWCNTs and SWCNTs structures were used. CNTs were either deposited as a coating on top of metal electrodes (Keefer et al., 2008; Gabriel et al., 2009; Fuchsberger et al., 2011) or directly grown from a catalyst patterned substrate (Wang et al., 2006; Gabay et al., 2007; Yu et al., 2007).

MWCNT-gold coated indium-tin oxide MEAs were used to record and stimulate mice cortical cultures by Keefer and co-workers. The CNT coated electrodes were found to be suited for recording and improved the effectiveness of stimulation (Keefer et al., 2008). Pristine CNT coatings were also used. Gabriel et al. coated standard platinum MEAs with SWCNTs which were directly deposited onto electrodes by drop coating and drying. CNT coating resulted with enhanced electrical properties, decreased impedance and increased capacitance. The researchers successfully performed extracellular recordings from ganglion cells of isolated rabbit retinas (Gabriel et al., 2009). Fuchsberger and co-workers proposed the deposition of MWCNT layers onto TiN microelectrode arrays by means of a micro-contact printing technique using PDMS stamps. The coated MEA was applied for the electrochemical detection of dopamine and electrophysiological measurements of rat hippocampal neuronal cultures. MWCNT coated microelectrodes were found to have recording properties superior to those of commercial TiN microelectrodes (Fuchsberger et al., 2011). Drop coating and micro-contact printing methods are quite simple to impalement. However, the film may have weak adhesion to the surface compared with covalent or electrochemical techniques, therefore careful validation of the coating adhesion is important.

**Figure 5:**
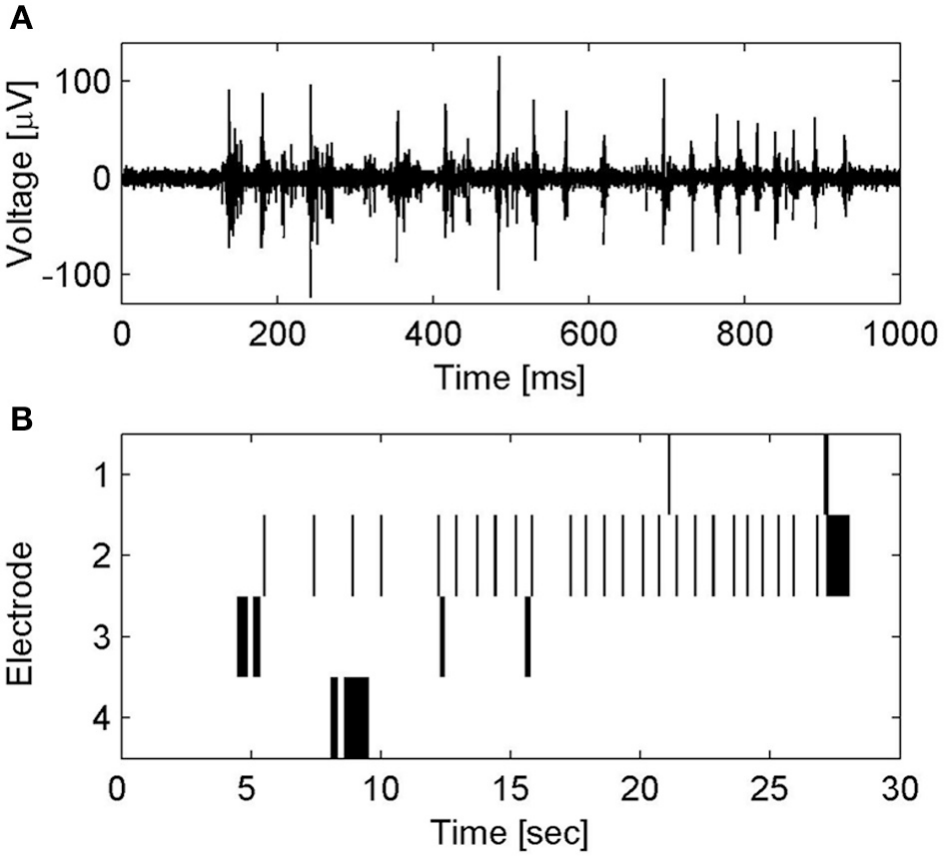
**Spontaneous electrical activity of neuronal clusters on CNT MEA. (A)** Voltage traces of spontaneous electrical activity recorded from a CNT electrode. **(B)** Raster plot of the spontaneous spiking activity in several CNT electrodes. Activity patterns are characterized by bursting events; short time windows (several hundreds of milliseconds) of rapid collective neuronal firing, which are followed by long intervals (seconds) of sporadic firing. For further details see Shein et al. (2009).

CNT MEAs based on top–down fabrication approaches were also reported. Superior electrical properties of CNT microelectrodes were presented by Gabay and co-workers. We fabricated the CNT MEAs by synthesizing high density MWCNT islands on a silicon dioxide substrate. The three-dimensional nature of the CNT electrodes contributes to a very large surface area, and consequently to high electrode specific capacitance (non-Fradaic behavior was validated) and low frequency dependence of the electrode impedance. Spontaneous activity of rat cultured neurons was recorded (Gabay et al., 2005a,b, 2007). Direct electrical interfacing between pristine CNT microelectrodes and rat cultured neurons was also demonstrated by Shein et al. (2009). Each electrode recorded the activity from a cluster of several neurons; this activity was characterized by bursting events (see Figure 5). The same CNT MEAs were further used to study the electrical activity of neuronal networks (Shein Idelson et al., 2010) as well as to interface with mice retina (Shoval et al., 2009). The retina tests revealed that SNR of CNT electrode improved over time suggesting a gradual (over 2 days) improvement in the tissue-electrode coupling. Recent stimulation tests by the same group revealed a similar improvement in the stimulation threshold (Eleftheriou et al., 2012).

Wang and co-workers presented a prototype of vertically aligned MWCNT pillars as microelectrodes on a quartz substrate (Wang et al., 2006). The nanotubes were functionalized with PEG to create a hydrophilic surface. The obtained hydrophilic CNT microelectrodes offer a high charge injection limit without Faradic reactions. *In vitro* electrical stimulation of embryonic rat hippocampal neurons was then achieved and detected by observing intracellular calcium level change using a calcium indicator (Wang et al., 2006). VACNF MEA was fabricated and tested for potential electrophysiological applications by Yu et al. (2007). Extracellular stimulation and recording of both spontaneous and evoked activity in organotypic hippocampal slices was reported. de Asis and co-workers systematically compared PPy-coated VACNF MEA with tungsten wire electrodes, a planar platinum MEA, and an as-grown VACNF MEA for the recording of evoked signals from acute hippocampal slices (de Asis et al., 2009). Recently Su and co-workers synthesized CNTs on a cone-shaped silicon tip by catalytic thermal CVD. Oxygen plasma treatment was used to modify the CNT surface to change the CNT surface characteristics from hydrophobic to hydrophilic in order to improve CNT wettability and electrical properties. Electrochemical characterization of the oxygen plasma-treated three dimensional CNT probes revealed lower impedance and higher capacitance compared with the bare silicon tip. Furthermore, the oxygen treated CNT probes were employed to record signals of a crayfish nerve cord (Su et al., 2010).

The development of CNT MEAs has a few important advantages over silicon probes commonly used in current neuroscience research and clinical applications. Silicon probes typically consist of a silicon support, silicon nitride, and silicon dioxide insulation layer. The electrodes are usually coated with iridium, gold or platinum. The first designs include the Michigan array (Wise et al., 1970; Wise and Angell, 1975) and the Utah array (Campbell et al., 1991). The Michigan probe includes several microelectrode sites patterned on each shank of the structure and the Utah array is a three-dimensional electrode array which consists of multiple sharpened silicon needles. However, a major shortcoming of these devices is the electrode material which is metallic and therefore Faradaic (compared with the capacitive CNT electrodes) and has no affinity to neuronal cells compared with the preferred neuronal adhesion to the rough CNT surfaces.

### Flexible CNT MEA for recording and stimulation of neuronal activity

Typical MEMS electrodes, despite their many advantages, are rigid and therefore are poorly suited for long-term neural *in vivo* applications. Accordingly, there is an increased interest in the development of flexible MEAs. Specifically, the combination of flexible substrates and CNTs electrodes for neuronal applications has gained attention.

Lin and co-workers were the first to fabricate and implement a flexible CNT-based electrode array for neuronal recording. The CNT electrode array was grown and patterned on a silicon substrate and was then transferred onto a flexible Parylene-C film. The four-step process included: CNT growth, polymer binding, flexible film transfer, and partial isolation. The resulting vertically aligned CNTs were partially embedded into the polymer film. Recording the electrophysiological response of a crayfish nerve cord was performed with two teflon-coated silver-wires used as a stimulation and a reference electrode. The SNR of the flexible CNT electrode was 257 (Lin et al., 2009).

Direct growth of CNTs on flexible polyimide substrates by catalyst-assisted CVD was also demonstrated (Hsu et al., 2010). The length of the MWCNTs was controlled and increased approximately linearly with the growth time resulting with decreased impedance and increased capacitance. UV-ozone exposure improved the interfacial properties between the CNT electrodes and the electrolyte by increasing the surface wettability (changing it from super hydrophobic to hydrophilic). UV-ozone treatment yielded a 50-fold impedance reduction. Furthermore, flexible CNT electrodes were found to exhibit resistive characteristics, in contrast to the results described above (Nguyen-Vu et al., 2006) which suggested that capacitive conduction dominates. Examination of neuronal cell cultures indicated good biocompatibility. Finally, recordings of evoked action potential from lateral giant neurons in the abdominal ganglia of crayfish were achieved. SNR was about 150, as good as that of a suction pipette and better than gold electrodes (SNR of 122 and 36, respectively). In a subsequent study, a flexible CNT MEA integrated with a chip containing 16 recording amplifiers was presented (Chen et al., 2011b). CNTs were again grown directly on a polyimide flexible substrate. The CNT microelectrode had ten times lower electrode impedance and six times higher capacitance, resulting with better charge injection capacity compared with a gold microelectrode of the same size. Tests with cultured neurons validated the biocompatibility of the device. *In vitro* spontaneous spikes were recorded from a caudal photoreceptor from the tail of the crayfish neuron with SNR of 6.2. The flexible CNT MEA was also applied to record the electrocorticography (ECoG) of a rat motor cortex.

**Figure 6:**
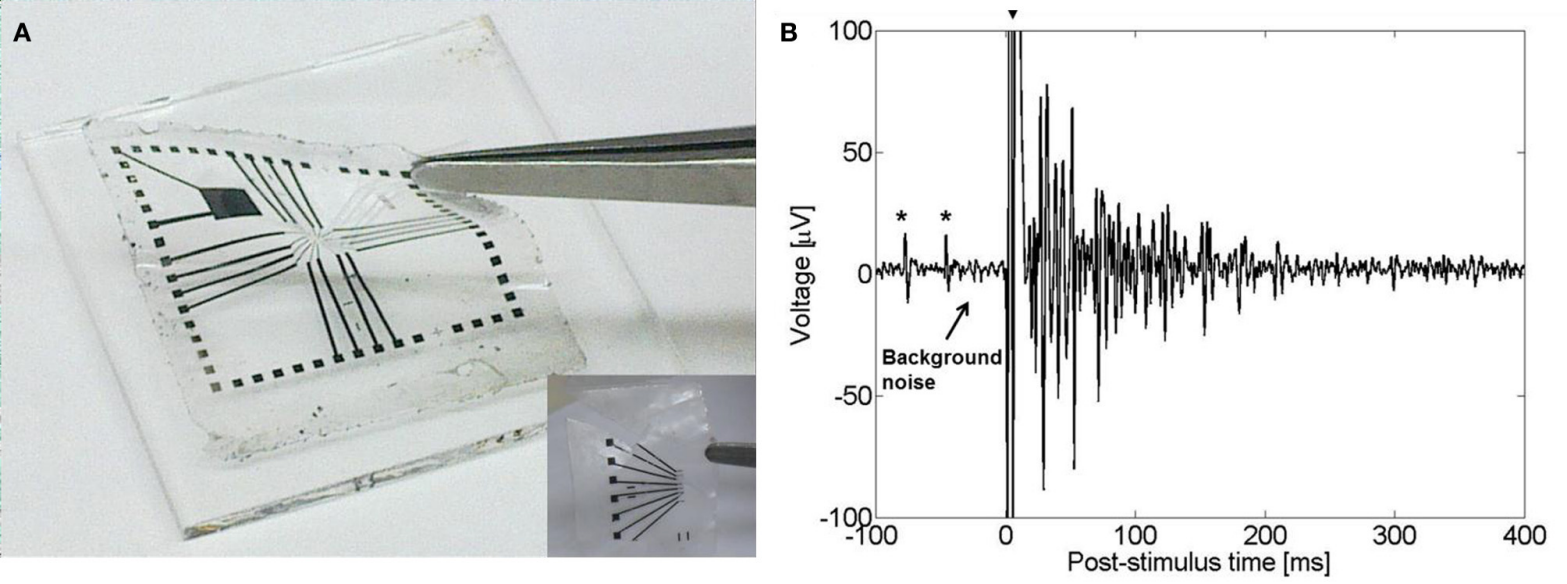
**(A)** A flexible CNT-based MEA. Inset: flexible CNT-based MEA designed for *in vivo* applications. (**B**) Evoked electrical activity recorded from an embryonic chick retina (day 14) by a CNT electrode (one out of sixteen 50 μm diameter electrodes in the array) using a biphasic anodic first pulse of 20 nC. Retina was flattened on the flexible CNT MEA with retinal ganglion cells layer facing down. The large signal at *t* = 0 (marked with arrow) is an artifact of the stimulation. Spontaneous activity prior to stimulation is marked with asterisks.

Our group has recently developed a novel all-CNT flexible electrode suited for recording and stimulation of neuronal tissue. Flexible devices were realized by transferring high density MWCNT films onto a flexible PDMS film (Hanein, 2010). A deliberate poor adhesion between the CNT film and the substrate allowed the transfer of the CNTs to the PDMS substrate (Figure 6A). This poor adhesion resulted from direct growth of the CNTs on SiO_2_. The technology is simple and the resulting stimulating electrodes are nearly purely capacitive. The electrodes exhibit a capacitance of 2 mF/cm^2^ which is similar to that of TiN and pristine MWCNTs electrodes fabricated on a rigid silicon substrate with 2 and 10 mF/cm^2^, respectively (Gabay et al., 2007). Recent recording and stimulation tests with chick retina (Figure 6B) validate the device suitability for high-efficacy neuronal stimulation applications (David-Pur et al., submitted).

Table 2 summarizes the main findings related to CNT-based neuronal electrical recording and stimulation. The overall picture that emerges from these data is that CNTs were used for neuronal electrical interfacing in three main schemes: CNT coated substrates, CNT coated sharpened wire metal electrodes and CNT MEAs. CNT substrates were used as an *in vitro* growth substrate for neurons and the electrical activity was recorded using intracellular patch clamp technique. Electrical stimulation through these CNT coated surface was also demonstrated. CNT coated sharpened wire electrodes were used for both *in vitro* and *in vivo* neuronal extracellular recording and stimulation. The CNT MEA scheme allows for *in vitro* patterned neuronal growth in conjugation with extracellular recording and stimulation. The final and most recent scheme is the development of flexible CNT MEAs which represents a major step toward implantable neuro-prosthetics applications.

**Table 2:** **Neuronal electrical interfacing CNT technologies**.

	**References**	**Electrode/surface description**	**Bio-testing scheme**	**Electrical recording**	**Electrical stimulation**
**CNT substrates**
	Lovat et al., 2005	MWCNTs coated cover slip	*In vitro* rat hippocampal cultures	Intracellular patch clamp	Not applied
	Gheith et al., 2006	SWCNTs-PAA coated cover slip	*In vitro* NG108 cell line	Intracellular patch clamp	Intracellular patch clamp
	Liopo et al., 2006	4-tertbutylphenyl or 4-benzoic acid modified SWCNTs coated PET film	*In vitro* NG108 cell line	Intracellular patch clamp	Extracellular
			*In vitro* rat peripheral neurons		
	Mazzatenta et al., 2007	SWCNTs coated cover slip	*In vitro* rat hippocampal cultures	Intracellular patch clamp	Extracellular
	Cellot et al., 2009, 2011	MWCNTs coated cover slip	*In vitro* rat hippocampal cultures	Intracellular patch clamp	Intracellular patch clamp
**CNT electrodes**
	Keefer et al., 2008	PPy-MWCNTs, Au-MWCNTs coated sharpened wire metal electrode	*In vitro* mice frontal cortex cultures	Extracellular	Extracellular (*in vitro* tests)
			*In vivo* rats motor cortex		
			*In vivo* monkeys visual cortex area		
	Baranauskas et al., 2011	PPy-MWCNTs coated sharpened wire metal electrode	*In vivo* rat cortex	Extracellular	Not applied
**CNT MEAs**
	Wang et al., 2006	Vertically aligned MWCNT MEA directly grown on a catalyst patterned substrate and coated with PEGPL for hydrophilic surface	*In vitro* embryonic rat hippocampal neurons	Not applied	Extracellular
	Gabay et al., 2007	MWCNT MEA directly grown on a catalyst patterned substrate	*In vitro* rat cortical cultures	Extracellular	Not applied
	Yu et al., 2007	VACNF MEA directly grown on a catalyst patterned substrate	*In vitro* organotypic rat hippocampal slices	Extracellular	Extracellular
	Keefer et al., 2008	ITO MEA (CNNS) coated with MWCNTs-Au	*In vitro* mice frontal cortex cultures	Extracellular	Extracellular
	de Asis et al., 2009	VACNF MEA directly grown on a catalyst patterned substrate and coated with PPy	*In vitro* rat hippocampal slices	Extracellular	Extracellular
	Gabriel et al., 2009	Pt MEA coated with SWCNTs (drop coating)	*In vitro* isolated rabbit retina	Extracellular	Not applied
	Su et al., 2010	Cone-shaped Si MEA coated with MWCNTs (CVD) after oxygen plasma treatment	*In vitro* crayfish giant neurons	Extracellular	Not applied
	Fuchsberger et al., 2011	TiN MEA coated with MWCNTs (micro-contact printing).	*In vitro* rat postnatal hippocampal cultures	Extracellular	Not applied
**Flexible CNT MEAs**
	Lin et al., 2009	Vertically aligned CNT MEA embedded in Parylene-C film	*In vitro* crayfish nerve cord	Extracellular	Not applied
	Hsu et al., 2010; Chen et al., 2011b	CNT MEA directly grown on a catalyst patterned polyimide after UV-ozone treatment	*In vitro* crayfish giant neurons	Extracellular	Not applied
			*In vitro* crayfish caudal photoreceptor		
			*In vitro* EcoG of rat motor cortex		
	David-Pur et al., submitted	all CNT MEA on PDMS	*In vitro* chick retina	Extracellular	Extracellular

## Conclusions and perspectives

In this review we explored the different properties that make CNT uniquely suited for neuronal interfacing. We have also shown that intensive investigations over the past 10 years have explored CNTs for neuronal interfacing, from surface properties effecting cell adhesion and proliferation to the development of CNT-based MEAs and flexible electrode arrays for *in vivo* applications. This intensive research was motivated by the need to find therapies for neural disorders which require the use of electrical stimulation, as well as by the need to address basic questions in neuroscience. In particular, the study of engineered neuronal circuits can greatly benefit from such CNT-based platforms. Neuronal circuits study aims at rebuilding damaged neuronal tissues. Natural circuits are not prone to manipulations and have highly complex structure and thus are extremely challenging to study. Engineered *in vitro* neuronal networks, however, allow monitoring and systematic investigation and provide unique platform for the study of activity patterns, morphology-activity relationship as well as network damage and repair methods. All These applications can greatly benefit from an efficient neuronal scaffold having the ability to record and stimulate neuronal electrical activity.

The challenging requirements in the field of neural prosthetics, namely, reduction of electrode size while maintaining efficient electrochemical function, as well as reduction of immune response to the implanted device (linked to both size and rigidity of the implanted device), are only poorly fulfilled by commonly used materials. Thus, the development of an efficient neuro-prosthetic platform will highly benefit from the realization of CNT electrodes on a flexible substrate.

The emerging applications of CNTs in the field of neuroscience must take into account cytotoxicity considerations. The potential toxicity of CNTs was extensively studied and so far revealed mixed results (Shvedova et al., 2003, 2009, 2010; Dumortier et al., 2006; Firme and Bandaru, 2010; Zhao and Liu, 2012). Better understanding of the interaction between CNTs and the biological environment is required in order to facilitate efficient development of both safe and effective CNT-based neural technologies. Further testing of CNT electrodes corrosion resistance as well as stress durability is required. Another essential step is further study of the nature of neuron-CNT electrical interfacing. Also, comprehensive long-term recording and stimulation studies in animal models followed by clinical trials and approval by administrative authorities such as the US food and drug administration (FDA) must be accomplished to allow routine use of CNT MEAs in neuroscience. The vast literature reviewed here, along with recent studies using CNTs embedded in polymeric support; show that CNTs, if handled properly, are safe as an implantable coating.

Several very promising directions in the study of CNT-based neuro-prosthetic devices currently exist: First is the integration of drug elution coatings. These coatings will allow the reduction of inflammation caused by the insertion of the neuronal implant to the tissue and improve survival of neurons in contact with the device. There is a growing interest in the study of such coatings (Zhong and Bellamkonda, 2005; Wadhwa et al., 2006; He et al., 2007), such studies will also benefit from addressing the development of a coating that will not impair the electrical activity of the device. Second, is the research toward realization of CNT-based flexible MEAs as elaborated in the text above. Some very recent work done in this area by our group and others revealed great potential of such devices. Finally, combining light-sensitive function with the enhanced neuronal interfacing properties of CNTs will be highly beneficial for the development of novel retinal implants.

To conclude, CNT enhanced electrochemical properties, their flexible and simple micro-fabrication preparation procedure, as well as their bio-compatibility and durability, suggest that CNT electrodes are a promising platform for high resolution neuronal applications. The resemblance of CNT surfaces to the nanostructured features of natural neural tissue makes CNTs a suitable platform for tissue engineering and regeneration (Tran et al., 2010; Voge and Stegemann, 2011). Also, the high electrical conductivity of CNTs allows direct electrical interfacing with neurons (Shein-Idelson et al., 2011). Clearly, CNTs have enormous potential in the development of neuronal interfaces and further study will enable the utilization of CNT-based technology to expand the understanding of the nervous system and for the realization of therapeutic approaches.

### Conflict of interest statement

The authors declare that the research was conducted in the absence of any commercial or financial relationships that could be construed as a potential conflict of interest.
